# Sustaining a New Model of Acute Stroke Care: A Mixed-Method Process Evaluation of the Melbourne Mobile Stroke Unit

**DOI:** 10.34172/ijhpm.2023.7716

**Published:** 2023-04-05

**Authors:** Kathleen L. Bagot, Tara Purvis, Shaun Hancock, Henry Zhao, Skye Coote, Damien Easton, Bruce CV Campbell, Stephen M. Davis, Geoff A. Donnan, Shane Foster, Francesca Langenberg, Karen Smith, Michael Stephenson, Stephen Bernard, Sharon McGowan, Bernard Yan, Peter Mitchell, Sandy Middleton, Dominique A. Cadilhac

**Affiliations:** ^1^Public Health and Health Services Research, Stroke, The Florey Institute Neuroscience and Mental Health, Heidelberg, University of Melbourne, Melbourne, VIC, Australia; ^2^Stroke and Ageing Research, Department of Medicine, School of Clinical Sciences at Monash Health, Monash University, Clayton, VIC, Australia; ^3^Department of Neurology, Melbourne Brain Centre, Royal Melbourne Hospital, Melbourne, VIC, Australia; ^4^Department of Medicine, Faculty of Medicine, Dentistry and Health Sciences, University of Melbourne, Melbourne, VIC, Australia; ^5^Austin Health, Faculty of Medicine, Dentistry and Health Sciences, University of Melbourne, Melbourne, VIC, Australia; ^6^Ambulance Victoria, Melbourne, VIC, Australia; ^7^Stroke Foundation, Melbourne, VIC, Australia; ^8^Department of Epidemiology and Preventive Medicine, Department of Paramedicine Monash University, Melbourne, Melbourne, VIC, Australia; ^9^Discipline of Emergency Medicine, University of Western Australia, Perth, WA, Australia; ^10^St Vincent’s Health Network Sydney, St Vincent’s Hospital Melbourne, Melbourne, VIC, Australia; ^11^Nursing Research Institute, Australian Catholic University, Melbourne, VIC, Australia

**Keywords:** Stroke, Ambulances, Evaluation Studies, Program Sustainability

## Abstract

**Background:** Internationally, Mobile Stroke Unit (MSU) ambulances have changed pre-hospital acute stroke care delivery. MSU clinical and cost-effectiveness studies are emerging, but little is known about important factors for achieving sustainability of this innovative model of care.

**Methods:** Mixed-methods study from the Melbourne MSU (operational since November 2017) process evaluation. Participant purposive sampling included clinical, operational and executive/management representatives from Ambulance Victoria (AV) (emergency medical service provider), the MSU clinical team, and receiving hospitals. Sustainability was defined as ongoing MSU operations, including MSU workforce and future model considerations. Theoretically-based on-line survey with Unified Theory of Acceptance and Use of Technology (UTAUT), Self Determination Theory (SDT, Intrinsic Motivation), and open-text questions targeting barriers and benefits was administered (June-September 2019). Individual/group interviews were conducted, eliciting improvement suggestions and requirements for ongoing use. Descriptive and regression analyses (quantitative data) and directed content and thematic analysis (open text and interview data) were conducted.

**Results:** There were 135 surveys completed. Identifying that the MSU was beneficial to daily work (β=0.61), not experiencing pressure/tension about working on the MSU (β=0.17) and thinking they did well working within the team model (β=0.17) were significantly associated with wanting to continue working within the MSU model [R^2^=0.76; F(15, 60)=12.76, *P*<.001]. Experiences varied between those on the MSU team and those working with the MSU. Advantages were identified for patients (better, faster care) and clinicians (interdisciplinary learning). Disadvantages included challenges integrating into established systems, and establishing working relationships. Themes identified from 35 interviews were MSU team composition, MSU vehicle design and layout, personnel recruitment and rostering, communication improvements between organisations, telemedicine options, MSU operations and dispatch specificity.

**Conclusion:** Important factors affecting the sustainability of the MSU model of stroke care emerged. A cohesive team approach, with identifiable benefits and good communication between participating organisations is important for clinical and operational sustainability.

## Background

**Key Messages**

** Implications for policy makers**

- New models of care disrupt established individual and organisational health system processes and may need alignment with new features, including multi-organisational and multi-discipline communication platforms, and sharing of pre-hospital medical information into hospital records.
- Factors important for ongoing operations (sustainability) can differ from those important for initial implementation, and need to be addressed separately within all involved parties.
- To support sustainability, the benefits to different stakeholders should be emphasised.
- Individual characteristics and team features are important for sustaining a new model of care and may influence the recruitment of staff or retention of the existing workforce.
- Technical capacity is an important factor to be considered for the individuals involved.

** Implications for the public**

Stroke is one of the leading causes of death and disability, with available treatments working best if delivered within the first 60 minutes of ischemic stroke symptoms starting. Fast diagnosis and treatment require specialist skills and equipment usually based in a hospital. Mobile stroke units (MSUs) are a specialised ambulance which brings a team of stroke experts and hospital equipment to the person with a suspected stroke. Having stroke experts working outside the hospital and paramedics working with them in the community is a new way of working for all these clinicians. Our research provides insights to support these changes. Specifically, it requires individuals from separate organisations to work closely together, to adapt usual ways of working to incorporate new team members, and to have good communication systems between the different team members and organisations. An MSU can offer rapid diagnosis and treatment options to members of the public experiencing this health emergency.

People who experience symptoms of stroke require rapid assessment and diagnosis so effective treatments can be offered to reduce the chances of disability or death.^1,2^ Traditionally, hyperacute stroke care includes use of ambulance services and triaging via emergency departments (EDs) to activate a ‘code stroke’ whereby a specialised team will assess the patient, diagnose and make treatment decisions.^3^ Precious minutes can be lost if the system is inefficient.^4,5^ A recent innovation to reduce assessment, diagnosis and treatment delays in stroke is through the use of specially designed ambulances known as mobile stroke units (MSUs).^6^ Since the first MSU was established in Germany in 2010,^7^ more than 30 active and planned MSU projects exist in various countries.^8^ These specialised ambulances house a computerised tomography (CT) scanner required for confirming a stroke diagnosis, point-of-care testing, and are staffed by a stroke-specialist team (eg, neurologist, stroke nurse^9^), a radiographer and paramedic personnel.^10^

 The advantages of MSUs to usual care have extended beyond early diagnosis and treatment delivery,^7^ improved treatment rates,^11^ more streamlined patient transport to appropriate hospitals for stroke and non-stroke neurological conditions^12^ and improved patient outcomes with pre-hospital treatment.^13^ MSUs can facilitate clinical trials of ultra-early stroke treatments.^14^ There is also some preliminary evidence that MSUs are cost-effective.^15-17^ The literature to date has predominantly focused on the clinical outcomes of MSUs. We have identified one recent review^10^ whereby the authors included a summary of the requirements for establishment and implementation of MSU programs; such details were available in only four of 38 included papers. Important steps detailed for implementing the Houston MSU included leadership, funding, legal requirements being met including insurance, multidisciplinary staffing and clinical protocols.^12^ In our research on the pre- and initial 24 month operations of the Melbourne MSU,^18^ we identified that despite the strong perceived benefits of working on the MSU being reported, a challenge was that the MSU workforce had to adjust to a variety of challenges (eg, workplace locations and culture). Concerns included limited resources such as only having one MSU in a city of 5.2 million people,^19^ and communication inefficiencies for sharing patient information between ambulance services and hospitals.^18^ To the best of our knowledge, there has been no research undertaken on the longer-term sustainability of MSUs, including maintaining the MSU workforce. As such, the aims of the current research were to describe and identify:

- Experiences across different healthcare professional groups working within the MSU model of healthcare for stroke;
- Perceived advantages (or benefits) and disadvantages (or barriers) of working within the MSU model;
- Factors associated with wanting to continue working within the MSU model;
- Factors to be considered for the long-term use of the MSU model.

## Methods

###  Study Design

 Within our retrospective, mixed-methods design, we used survey and interview methods to conduct our multi-stakeholder process evaluation of the Melbourne MSU.^20^ Factors specifically relevant to the pre-operational and initial (18-24 month) operational period are presented elsewhere.^18^ In this paper, we present the findings related to the questions explored on the issue of operational sustainability of the MSU as implemented within the context of Melbourne, Australia. The data from this study are drawn as a component of the larger process evaluation. This manuscript has been prepared in line with the consolidated criteria for reporting qualitative research (CORE-Q).^21^

###  Setting and Context

 The Melbourne MSU is the result of a multi-organisation collaboration between the Royal Melbourne Hospital (RMH), Ambulance Victoria (AV), the University of Melbourne, the Florey Institute of Neuroscience and Mental Health, the Stroke Foundation and RMH Neuroscience Foundation.^20^ Launched in November 2017, clinical outcomes^20^ alongside initial cost-effectiveness^15^ for the Melbourne MSU have been previously published, with brief details provided here. The MSU ambulance houses a CT scanner and is staffed with a multidisciplinary acute stroke team of five: a neurologist/senior stroke fellow, a stroke nurse, a CT radiographer and two paramedics (one advanced life support [ALS] and one mobile intensive care ambulance [MICA] paramedic). A MICA paramedic has a higher clinical skill set than ALS paramedics and can perform more advanced medical procedures. During operational hours (8 am to 6 pm, Monday to Friday), it is co-dispatched with a standard ambulance to suspected stroke cases within 12 hours of symptom onset and within a 20 km radius from RMH, via a dispatch matrix. The MSU can also self-dispatch to cases outside these parameters (up to 70 km within metropolitan Melbourne), and regular ambulance crews can request the MSU from anywhere in metropolitan Melbourne. Where appropriate, patients are assessed, diagnosed and treated in field. Patients with a stroke diagnosis are transferred to the nearest thrombolysis stroke centre or bypassed to the nearest comprehensive stroke centre, as relevant to treatment requirements. Pre-notification via radio between the ambulance team and the hospital ED team is standard practice for AV to receiving hospitals. With AV paramedics and hospital clinicians working in the field on the MSU, new and additional communication pathways between all parties were involved. These formal and informal pathways included between the dispatchers, to the co-dispatched ambulance paramedics and MSU paramedics, between co-dispatched paramedics and MSU team via the dispatcher, between MSU team clinicians and receiving hospital ED and/or to receiving hospital stroke team.

###  Participants

 Potential participants were identified by the MSU project manager and receiving hospital contacts (insider assistants), as deemed relevant to our aims (purposive sampling).^22^ These included individual members of the MSU project team, organisational executives, MSU clinical staff, and clinicians at receiving hospitals who were involved in managing MSU patients.

 Individuals across clinical, operational, executive, and project management roles involved in the development, implementation and operations of the MSU were invited to participate. Organisations included the Emergency Services Telecommunications Authority (ESTA; statutory authority responsible for all emergency service call taking and dispatch in Victoria, Australia, including emergency ambulance), and hospitals – MSU clinician-provider and MSU base hospital (RMH) and patient receiving hospitals (8 metropolitan, thrombolysis capable hospitals, including RMH). Therefore, data were collected from ESTA dispatchers, co-dispatched paramedics, paramedics and hospital clinicians who worked on the MSU, hospital clinicians at receiving hospitals, including emergency medicine, radiology, neurointerventional and neurology/stroke team, plus MSU program operational team members and executives across participating organisations.

###  Data Collection

 Data sources were a survey (maximum total of 49 items for the complete survey/comprehensive evaluation; Supplementary file 1, Figure S1) administered July to September 2019 and individual and group interviews (Supplementary file 1, Figure S2 for interview schedule) conducted during October and November 2019. Item details specific to this study addressing sustainability are presented here.

*Survey – *Demographics of role and organisation, time in role, age, gender, and employment status were requested (7 items). To identify aspects relevant for the sustainability of the MSU, potential factors impacting wanting to work on/with the MSU program in future were explored, drawing on three established theories and those developed specifically for the MSU evaluation (example items in Table 1, full list in Figure S1). Measures were selected to ensure coverage of potentially relevant factors of implementing and incorporating a new technology-based system into an established system. The items in each measure were adapted to target the MSU program, and included factors previously identified as influencing the acceptance and use of new technologies (measured with the unified theory of acceptance and use of technology [UTAUT] questionnaire) organisational factors such as readiness for implementing change (measured with the Organizational Readiness for Implementing Change [ORIC] questionnaire) and individual factors such as personal motivation to work on the MSU (measured with the Intrinsic Motivation scale of the Self Determination Theory [IM-SDT] questionnaire).

**Table 1:** Survey Item Source, Construct and Example Item

**Measure**	**Construct**	**Example Item/s**
UTAUT^23^	Behavioural intentions	“I would like to work on/with the MSU program in the future” (outcome variable for regression analysis)
	Performance expectancy	“Working on/with the MSU program was beneficial in my daily work”
	Effort expectancy	“My role in working on/with the MSU program was clear”
	Facilitating conditions	“I felt I had the resources necessary to work on/with the MSU program”
	Habit	“Working on/with the MSU program has become natural to me”
	Social influence	“Peers and managers were supportive of my role working on/with the MSU program”
ORIC^24^	Perceived organizational readiness	“I felt my team/organisation were ready to work on/with the MSU program”
IM-SDT^25^	Interest	“I found working on/with the MSU program interesting across entire program development”
	Perceived competence	“After working on/with the MSU program for a while, I felt competent in my role in relation to the program”
	Perceived choice	“I did not really have a choice about working on/with the MSU program”
	Pressure/tension	“I did not feel at all nervous about working on/with the MSU program as a new model of care”
Developed specifically for MSU evaluation	Impact on role	“My role has changed as a result of working on/with the MSU program” “I felt like I was able to work to my full scope of practice while I was working on/with the MSU program”

Abbreviations: MSU, mobile stroke unit; UTAUT, unified theory of acceptance and use of technology; ORIC, Organizational Readiness for Implementing Change; IM-SDT, Intrinsic Motivation scale of the Self Determination Theory.

 Ten items exploring individual clinicians’ acceptance and intentions to work with/on the MSU were drawn from the UTAUT.^23^ Each participant’s perceptions of their organisation’s readiness for change was measured using a single item from the ORIC^24^ measure. Individuals’ motivations to work on/with the MSU were examined with the IM-SDT^25^ (7 items). Two additional items were developed specifically for this study. Survey instructions were to review each statement and indicate the level agreement on a seven-point Likert-type response scale: 1 completely disagree to 7 completely agree. Response options also included “not relevant to me.” Higher scores indicated stronger agreement. To ensure item relevance for each participant, survey items presented varied depending upon the role of the participant. In addition, two open-text questions asking about the *benefits of* and the *barriers to* working on the MSU program were included.

 Email invitations including the online survey link were distributed by authors TP and DE (the MSU Project Manager). An invitation to all paramedics co-dispatched and call takers/dispatchers who had worked with the MSU to complete the survey was placed on internal sites – the intranet and Workplace (ie, the enterprise social network designed to support communication, collaboration and connectedness).

 Survey completion implied participant consent and were completed anonymously. However, participants could provide their name and contact details if they wished to participate in subsequent interviews, with details kept separate from survey response data. Reminders were circulated after 2-3 weeks. All surveys were administered and completed online using Qualtrics^TM^ (version XM).

*Individual and Group Interviews – *A purposive sample of those who completed the survey were invited to participate in individual or group interviews. A semi-structured, open-ended interview guide was used, focusing on operational and organisational aspects of the MSU. A series of questions targeting individual’s experiences with the MSU and opinions regarding its operations were undertaken. Responses were probed or prompts provided to ensure information provided was understood. The main question relevant to the current study was *Do you have any suggestions for improvements or requirements for future long-term use?* Probes included *rollout process, training requirements, information and education. *During the course of interviews, responses to other questions that led to reflections on future requirements were also included here.

 Individual interviews were face-to-face and by telephone (approximately 45-60 minutes), with group interviews held face-to-face (approximately 1.5 hours); all conducted by author TP (experienced qualitative researcher with stroke research expertise). Individual or group interviews were used pragmatically, as suited the participants availability. Participants were advised that their feedback and experiences were to improve the service planning and inform the development of future similar services. All interviews were audio-recorded, with participants’ permission. Recordings were transcribed verbatim and participants could review prior to analysis.

###  Data Analysis

 For the analysis, participants were grouped into three sub-samples: those working ‘on the MSU’ (clinical staff based on the MSU: paramedics, neurologist, radiographer, stroke nurse), those working ‘with the MSU’ (ESTA dispatchers, co-dispatched paramedics, clinicians at receiving hospitals), and the ‘MSU Project Team’ – those who were part of the MSU operational team or partner organisations (organisational executives and managers, program operational team including project managers and project leads; some of who also worked clinically on the MSU). Participants who worked on the MSU and were also involved in program operational roles were included in the MSU Project Team group given these wider operational roles may provide different insights to stand-alone clinical roles. Small numbers of operational and non-clinical team members of this sub-sample precluded examination as a separate group.

###  Survey Data

####  Quantitative Analysis

 Where multiple items were included for each construct, assessment of internal consistency was conducted with Cronbach’s alpha. If 0.7 or greater, items were pooled for analysis, if less than 0.7, individual items were retained. Analysis of variance was used to compare results across the three groups. Bivariate correlations were examined prior to conducting multivariable regressions with constructs of Performance Expectancy, Effort Expectancy, Facilitating Conditions, Habit, Social Influence, Perceived Organizational Readiness, Interest, Perceived Competence, Perceived Choice, Pressure/Tension, and Impact on role predicting participants wanting to work on/with MSU program in future (Behavioural Intention). Significant results will identify which of these factors is important for participants’ continued involvement with the MSU model. Analyses were undertaken in STATA (v16) and statistical significance was determined at *P*< .05.

####  Qualitative Analysis

 For survey open-text responses to questions seeking benefits (ie, advantages) and barriers (ie, disadvantages) of the MSU, a deductive approach was undertaken using directed content analysis.^26^ This systematic approach supports identifying themes within these short-answer responses and the frequency of thematic incidences can be reported. The UTAUT domains (on which the survey was based) were used as the coding framework, to support deeper understanding of the quantitative results. Responses that did not fall within the UTAUT framework were able to be coded separately, ensuring no data were missed. Two independent coders (SH, Honours, Psychology; KB, PhD, Psychology) reviewed and allocated all items to a sub-category, which were then grouped to form an overarching category within each UTAUT domain. Meetings between KB and SH enabled review and discussion of all categories and sub-categories, resulting in a refined final coding framework with all categories harmonised and renamed. Coding was undertaken within an Excel spreadsheet and inter-rater reliability calculated based on sub-category agreement.

####  Individual and Group Interview Data Synthesis

 For the interview data, descriptive thematic analysis was undertaken using an inductive approach (ie, no pre-defined framework was deployed; data-driven results).^27,28^ Established procedures were followed: read and re-read transcripts (phase 1 familiarises with data), line-by-line analysis of text and identification of initial categories (phase 2 coding) and subsequent grouping and hierarchical consideration of related categories (phase 3 generating initial themes). Important themes and sub-themes were refined and identified (phase 4 reviewing themes). One coder (KB, PhD, psychology) completed all initial coding and each theme/sub-theme was subsequently reviewed by authors TP (interviewer, BPhyiso(Hons), MSc) and DC (research principal investigator, PhD, public health) (phase 5 define and name themes). Final themes and sub-themes were endorsed by all authors (phase 6 write up). Illustrative quotes are provided verbatim, with spelling and grammar corrected.

 As relevant, the data from each aspect of this study were then synthesised to inform the summary of factors that influence sustainability of the Melbourne MSU (ie, triangulation).

## Results

 There were 135 surveys completed across the participant groups (Table 2).

**Table 2:** Participants Completing Surveys and Interviews/Focus Groups, by Organisation and Role

**Categorisation**	**Role**	**Survey**		**Interviews**	
		**n = 135**	**%**	**n = 38**	**%**
**Worked with MSU**	ESTA dispatchers	11	8	3	8
	AV paramedics	68	50	6	16
	ALS paramedic co-dispatched	49		4	
	MICA paramedic co-dispatched	18		1	
	MICA paramedic (Communications support paramedic - worked with ESTA call takers/dispatchers to support decisions about ambulance/MSU dispatch)	1		1	
	Clinicians from receiving hospitals^b^	17	13	11	29
	Pharmacist	1		1	
	Stroke nurse	7		3	
	Radiographer			1	
	Neurologist/stroke fellow/neurointerventionalist	9		6	
**Worked on MSU**^a^	AV paramedics	13	10	4	11
	ALS paramedic	7		1	
	MICA paramedic	6		3	
	Clinical staff	11^c^		3^d^	8
	Radiographer	4			
	Nurse	3		1	
	Neurologist/stroke fellow	4		2	
**MSU project team member**	Organisation executive or manager only (AV)	6	4	0	0
	Program operational team	9	7	11	29
	Melbourne Health	0	0	1	3
	AV	2		3	
	Also worked as paramedic on MSU	3		3	
	Also worked as clinical staff on MSU	4		4	

Abbreviations: ALS, advanced life support; ESTA, Emergency System Telecommunications Authority; MICA, mobile intensive care ambulance; MSU, mobile stroke unit; AV, Ambulance Victoria. Note: May not add to 100% due to rounding. ^a^Includes staff with no project team involvement; ^b^Excluding clinical staff from RMH also working on the MSU; ^c^ Including 7 staff from RMH who also referred to themselves as working for receiving hospitals; ^d^Including 2 staff from RMH who also referred to themselves as working for receiving hospitals.

 The majority of participants were paramedics co-dispatched with the MSU (50%), and we obtained at least one response from clinical staff at each receiving hospital. Participants had a median time in their role of 8 years (interquartile range: 2, 12.67).

###  Experiences Across the MSU Clinical and Operational Sub-samples

 Although there were significant differences between sub-samples (ie, groups) for agreement with statements, mean scores across the three groups were generally in the same area of the response scale (Table 3); that is, indicating all groups typically agreed (scoring on average above 4) or disagreed (scoring on average below 4) with the statements.

**Table 3:** Group Comparisons Between Those Working on the MSU, With the MSU and Leading the MSU Work

**Variable**	***P*** ** Value**	**Mean (SD)**		
		**On MSU** ***N Varies*** ^*^ *** 8-24***	**With MSU** ***N Varies*** ^*^ *** 77-89***	**Project Team** ***N Varies*** ^*^ *** 4-15***
Effort expectancy	.15	5.06 (1.03)	4.80 (1.13)	5.39 (1.15)
Facilitating conditions	**.02**	5.46 (0.73)^a^	4.90 (0.96)^a^	5.38 (0.99)
Perceived tension	**.004**	3.92 (1.20)^a,b^	3.00 (1.24)^a^	2.82 (1.25)^b^
Perceived organisational readiness	**.003**	5.54 (0.88)^a^	4.79 (1.20)^a,b^	5.57 (0.94)^b^
Working on/with the MSU program has become natural to me	**<.001**	5.67 (1.24)^a^	4.49 (1.40)^a,b^	5.75 (1.16)^b^
I would like to work on/with the MSU program in the future	**.02**	6.29 (0.95)^a^	5.33 (1.61)^a^	5.85 (1.34)
I found working on/with the MSU program interesting (across entire program development)	**<.001**	6.38 (0.97)^a^	5.33 (1.38)^a,b^	6.40 (0.99)^b^
I did not really have a choice about working on/with the MSU program	**<.001**	2.04 (1.66)^a^	4.64 (1.39)^a,b^	3.08 (1.88)^b^
I think I did well at working on/with the MSU program, compared to colleagues	.23	4.58 (1.18)	4.54 (1.08)	5.29 (1.11)
After working on/with the MSU program for a while, I felt competent in my role in relation to the program	**.003**	5.79 (0.93)^a^	4.95 (1.26)^a^	5.88 (0.99)
I felt like I was able to work to my full scope of practice while I was working on/with the MSU program	.27	4.58 (1.28)	4.78 (1.39)	5.50 (1.60)
Working on/with the MSU program was beneficial in my daily work	.17	5.75 (0.71)	4.81 (1.44)	4.50 (1.00)
Peers and managers were supportive of my role working on/with the MSU program	**<.001**	6.13 (0.94)^a^	5.08 (1.10)^a^	6.00 (1.07)
My role has changed as a result of working on/with the MSU program	**<.001**	4.25 (1.59)^a^	3.37 (1.38)^a.b^	4.47 (1.70)^b^

Abbreviations: MSU, mobile stroke unit; SD, standard deviation. Note: Working on MSU = paramedics working on the MSU, and hospital clinicians working on the MSU; working with the MSU = paramedic co-dispatched, ESTA or AV Communications Support Paramedic, and clinicians at receiving hospital; and leading MSU work = Executives, organisational managers, and program operational team (including program operational team that worked on the MSU). Bold indicates significant differences. ^*^ N varies due to missing data and presentation of items to relevant participants. Superscript with same letter denote significant differences between these groups identified with Tukey-Kramer post hoc analyses.

 All groups agreed that they would like to work on/with the MSU program in the future, that they found working on/with the MSU program interesting and beneficial to their work, felt competent in their role, were able to work to their full scope of practice and had their peer/manager’s support.

 There were two exceptions to this pattern: responses to ‘*I did not really have a choice about working on/with the MSU program’* and ‘*My role has changed as a result of working on/with the MSU program.*’ For the former question, those working with the MSU slightly agreed (on average scoring above the mid-point on scale of 4) and scored significantly higher than the other two groups (on the MSU and Project Team) who disagreed (on average scoring below the mid-point of 4) with not having a choice to work on/with the MSU program. Whereas for the latter question, those working with the MSU slightly disagreed with this item (on average just below mid-point of 4) and scored significantly lower than the other two groups who slightly agreed (on average just above mid-point of 4) their role had changed.

 Most group differences reported were those working with the MSU scoring significantly lower (approximately 1 point lower on 7-point scale) than those working on the MSU, and/or significantly lower than the Project Team group. This pattern included that those working with the MSU reported significantly lower Perceived Organisational Readiness and “Working on/with the MSU program has become natural to me.”

###  Participant Identified Advantages and Disadvantages of Working Within MSU Model

 From the open-text survey questions, there were 234 responses addressing advantages identified by 102 participants, and 228 responses addressing disadvantages reported by 94 participants, which were then grouped into themes and sub-themes (Table 4). Inter-rater coding agreement for advantages was 84% agreement (201/234) and 92% for disadvantages (209/228) at the theme level.

**Table 4:** Benefits and Barriers of Mobile Stroke Unit Model of Care

**UTAUT Framework** **n (% Of Comments)**	**Themes**		**Sub-themes**
		**Benefits/Advantages (N = 234)** **n (% Of UTAUT Domain)**	**Barriers/disadvantages (N = 228)** **n (% Of UTAUT Domain)**
Facilitating conditions Benefits 74 (32%) Barriers 122 (54%)	Availability	NA	Limited operating hours, area, only one MSU, 47 (39%)
	Communication	Between MSU, AV, pre-hospital, and specialist handover, 6 (8%)	Suboptimal handover/communication between MSU and co-dispatched paramedics or receiving hospital, no unified communication system for all stakeholders, 18 (15%)
	Resources	Funding available, 1 (1%)	Limited resourcing *no other details provided*, questionable if cost-effective, 2 (2%)
	Knowledge	Improved knowledge for neurology/stroke care, complete pathway including pre-hospital to stroke unit, learning environment, access to specialist knowledge, 53 (72%)	Not familiar with MSU operating protocol, MSU team roles, stroke-specific knowledge, limited exposure to MSU, unknown processes into established system, 32 (26%)
	Work environment	New, dynamic, exciting, challenging, no night shift, 14 (19%)	Very different, MSU paramedics on standby are in different environment to usual ambulance base (described by some as not appropriate), lack of ongoing training/education, 9 (7%)
	Vehicle	NA	Issues with design/build (eg, difficulty scanning in hot weather, motion sickness) workflow, communication, temperature control in truck, out of service periods, 14 (12%)
Performance expectancy Benefits 126 (54%) Barriers 66 (29%)	Skills	Improved co-dispatched paramedic skills – upskill, confidence, 3 (2%)	Repetitive nature of caseload, 1 (2%)
	Patient care	Efficient, faster, best practice, 9 (7%)	Delayed care, patient deterioration, waiting on scene, 4 (6%)
	Patient outcomes	Improved patient outcomes, 24 (19%)	Benefits yet to be seen, limited capability of MSU, 4 (6%)
	Patient-specific processes	Improved – faster/earlier diagnosis, treatment, triage, transfer decision making, faster scan, 53 (42%)	Integrating MSU into work practice – Location of patient/MSU/hospital precludes need for MSU, increase in AV on-scene metric, more patients to RMH = increase in bed pressure, time constraints, 40 (61%)
	Stroke care processes	Improved access to stroke specialist skills, advice, world class model, 37 (29%)	Increased workload – Competing non-MSU role demands, radio traffic increased, long hours/driving, dispatch specificity, 17 (26%)
Social influence Benefits 34 (15%) Barriers 29 (13%)	Processes	Project management, 2 (6%)	Rostering across year, 1 (3%)
	Working relationships – individual/discipline level	Interdisciplinary interactions, working with other health professionals, 16 (47%)	Lack of autonomy, 1 (3%)
	Working relationships – team level	Working with a great team, 6 (18%)	Unclear role delineation, inconsistency in approach, juggling pre-hospital generic vs stroke specific care, large number at scene, different interpretation of requirement for MSU based on initial paramedic scene situation report, some of MSU crew attitude, 23 (79%)
	Working relationships – organisational level	Collaboration, 10 (29%)	Too many stakeholders, power imbalance, not fully supported, 4 (14%)
Unclear comments	Unclear	0	9

Abbreviations: AV, Ambulance Victoria; MSU, mobile stroke unit; NA, not applicable; RMH, Royal Melbourne Hospital; UTAUT, unified theory of acceptance and use of technology. Note: May not add to 100% due to rounding, additional unclear comments.

 Advantages were mostly associated with performance expectancy (53% of identified advantages associated with job performance enhancements), and included improved patient-specific processes (eg, faster diagnosis and treatment times, improved triage and transfer decision making), improved patient outcomes (non-specific) and systemic level improvements through improved stroke care journey processes (eg, improved access to stroke specialist skills and advice, world class service). Categories within facilitating conditions (31% of identified advantages associated with infrastructure required to support use) included improved neurology/stroke knowledge (assessment, diagnosis, treatment), greater understanding of entire stroke care pathway, and a positive learning environment for both clinicians and paramedics. Advantages within the social influence domain (15% of advantages associated with important others) included working relationships at individual/discipline (eg, interdisciplinary interactions), working with a great team and collaboration across organisations.

 Advantages were reported by all groups. Those who worked with the MSU identified advantages mostly with improved patient-specific care processes and improved stroke care journey processes while those who worked on the MSU identified advantages mostly with improved knowledge and improved patient-specific care processes.

 Disadvantages were mostly associated with facilitating conditions and included availability of the MSU (eg, operating hours, area of operation, only one MSU available), lack of knowledge (eg, first in Australia, not familiar with MSU protocol) and unclear roles when working with the MSU team. Social influence included working relationships, and having to juggle roles focused on pre-hospital care with stroke care specifically.

 While disadvantages were reported by all groups, most of these were identified by those who worked with the MSU. Notably, there were many unique, specific disadvantages raised by single individuals, indicating the diverse experiences of those involved.

###  Predicting Wanting to Work on/With the MSU Program in Future 

 Examining the bivariate correlation matrix, most variables were significantly correlated, and no multi-collinearity was identified. However, a positive correlation in the bivariate results was negative in the multivariable regression; that is, suppression^29^ was identified. The item *I think I did well at working on/with the MSU program, compared to colleagues* became a negative predictor once other variables were included in the regression.

 There were three significant factors associated with wanting to work on the MSU in future (Table 5; R^2^ = 0.76; F(15, 60) = 12.76, *P*< .001).

**Table 5:** Regression Analysis Summary for Predicting Intentions to Work on or With the MSU in the Future (n = 76)

**Variable**	**B**	**B SE**	***P *** **Value**	**β**
Group (compared to working on the MSU)				
With the MSU	-0.36	0.44	.42	-0.08
Project Lead (including project leads who worked on the MSU)	0.71	0.68	.30	0.08
Effort expectancy	-0.19	0.16	.25	-0.14
Facilitating conditions	-0.06	0.17	.74	-0.03
Perceived tension (SDT)	**-0.24**	**0.12**	**.04**	**-0.19**
Perceived organisational readiness	0.02	0.15	.91	0.01
Habit – Working on/with the MSU program has become natural to me	0.18	0.11	.10	0.16
Interest – I found working on/with the MSU program interesting (across entire program development)	0.15	0.11	.17	0.13
Perceived choice – I did not really have a choice about working on/with the MSU program	0.07	0.07	.38	0.08
Perceived competence – I think I did well at working on/with the MSU program, compared to colleagues	**-0.23**	**0.11**	**.03**	**-0.17**
Perceived competence – After working on/with the MSU program for a while, I felt competent in my role in relation to the program	0.12	0.10	.22	0.11
I felt like I was able to work to my full scope of practice while I was working on/with the MSU program	0.04	0.12	.77	0.03
Performance expectancy – Working on/with the MSU program was beneficial in my daily work	**0.69**	**0.11**	**<.001**	**0.61**
Social influence – Peers and managers were supportive of my role working on/with the MSU program	0.10	0.15	.48	0.08
My role has changed as a result of working on/with the MSU program	0.02	0.07	.77	0.02

Abbreviations: SE, standard error; SDT, Self Determination Theory; MSU, mobile stroke unit. *Note*: Bold where *P* < .05. Variance inflation factor < 4 for all variables; no collinearity.

 The three factors were: (*i*) working on/with the MSU was beneficial in their daily work, (*ii*) not feeling pressure/tension about working on MSU, and (*iii*) not making comparisons that they worked better than other colleagues (ie, perceived as working as part of a team).

###  Future of Mobile Stroke Unit From Interviews

 There were 23 individual and 5 group interviews (with 2 to 4 participants in each; 2 groups of clinicians at receiving hospitals, 1 group each of paramedics co-dispatched with MSU paramedic, dispatchers, clinicians on MSU) conducted, totalling 38 participants. Three of these participants had not completed the survey, however, were directly approached by author TP to be interviewed as they were deemed to be key project staff. Seven themes covered aspects of future MSU operations (Table 6).

**Table 6:** Summary of Future Considerations for a Mobile Stroke Unit Program

**Theme**	**Detailed Sub-theme/Consideration**
MSU team model	Number rostered on the MSU: size of MSU needs to be considered to accommodate a minimum number of rostered team members who have different roles and responsibilities.
	Staff configuration: need to ensure all essential staff are on-board. The minimum would be at least one MICA paramedic, a radiographer and a stroke nurse specialist with access to a neurologist via telehealth. Some task substitutions could occur if supported by training, policies and considered within relevant scopes of practice.
Recruitment/rostering	Concerns regarding deskilling (ie, paramedics and radiographers) as the scope of practice is narrower on an MSU for some clinical staff which can be overcome through ensuring regular rotation of staff where feasible.
	The remuneration and opportunities to earn the same wages as when working in other roles is important (eg, out-of-hours allowances).
	Need to ensure access to a highly skilled workforce that can meet the rostering requirements for the MSU (eg, there may be insufficient MICA trained paramedics, stroke neurologists or highly skilled stroke nurse practitioners).
	Core team for each rostered day: preference was to have the same core team for a rostered day rather than having split shifts over the one day for radiographers (two shifts per day) to maintain team dynamics.
Multi-organisation communication	Systems for effective internal communication, training and professional development within the Ambulance service for those working on the MSU or standard ambulances that are to be co-dispatched to ensure relevant information about the MSU or potential MSU patients reaches the staff involved.
	Each receiving hospital to be involved in optimising the MSU patient transfer flow, and communication and documentation processes for their individual hospital systems.
	MSU patient numbers and patient outcome feedback, including catchment details, to be circulated to receiving hospitals to keep them engaged and informed.
Telemedicine	Telemedicine infrastructure and processes can be difficult to implement for a variety of reasons (concerns include the reliance on technology; telemedicine link potentially dropping out at critical times and not having a neurologist on board MSU for patient care) and barriers need to be overcome for this type of model to be successfully adopted for an MSU service.
	To ensure adequate coverage of neurologists for the MSU it would be an advantage to link an MSU service with an established acute stroke telemedicine service to ensure coverage and access to those with experience and credentialing.
	Future MSU ambulances to be built with state-of-the art and reliable technology (eg, inbuilt with a dedicated WIFI hub to support remote clinical decision-making via telemedicine protocols).
Investment in MSUs	The costs and benefits of investing in MSUs needs to be assessed from a financial and economic perspective, including considerations of economies of scale if more than one can be justified within a geographical location. The feasibility of operating these services needs to also consider whether there is access to skilled and expert health professionals as outlined in the sections above.
	Numbers of patients that will be treated needed to be estimated as part of business cases to determine whether the investment is worthwhile in terms of effectiveness and cost-effectiveness.
	Scope of medical services provided on the MSU: feasibility of investment should also consideration expanding the potential services offered; for example, ability to treat cases with hyperglycemia or manage paediatric cases.
Dispatch	Ensure the dispatch protocol will be configured to optimise stroke case identification.
	Review co-dispatch protocol and determine whether ALS ambulance needs to be co-dispatch each time.
	Coverage: ensure available for 24/7 dispatch.
Vehicle/s	MSU truck design is suitable including size, technology features, and interior layout to support optimal functionality and staff comfort in performing their roles.
	A single truck is not ideal to ensure continuity of service and equity of access to the service. Additional trucks are required to increase the geographical areas that the MSU can reach and ensure availability of the service (eg, when one truck is requiring maintenance there is still a truck working).
	Location of the trucks: not hosted in the same location to ensure appropriate coverage and equitable access within the defined geographical service boundaries. Requires careful consideration as there will be a range of factors to consider including whether the additional MSU vehicle/s are to work with the primary MSU (eg, as a back-up) or to work individually (eg, different area).
	Consideration of alternate vehicles or approaches (eg, stroke neurologist accessible by road with a standard car, not MSU).

Abbreviations: MSU, mobile stroke unit; ALS, advanced life support; MICA, mobile intensive care ambulance. Note: Themes and subthemes associated with the UTAUT dimension of facilitating conditions.

 Future considerations included aspects of the MSU vehicle (eg, number of vehicles, location of vehicles, changes to current vehicle). While there was little agreement as to where the best location was to house and run the current and any additional vehicles, physical changes to the MSU itself were often raised, particularly regarding communication.

 “*… I’d probably do a dual cab. I would eliminate the fact that we are separated … two paramedics at the front and three of them sitting in the back, we could all just have a generalised conversation like we’re in a normal car. And then we wouldn’t need an intercom, so that would eliminate the issues with talking. Everyone could hear the same dispatch, the same radio information, so we don’t have to relay stuff. So that would cut out a lot of that. So I think that would be, probably, the number one thing that I would change in the vehicle”* (Participant 06, Paramedic on MSU).

 The were mixed opinions about any future MSU team composition and roles (eg, neurologist off vehicle, duties of nurse and paramedics to be broadened/amended) moving forwards.

 “*Five people working on a unit is a lot. Clearly, you need a radiographer, as they are the only ones in this country that can take a picture using the radiation. I think beyond that you probably need a MICA paramedic and the nurse”* (Participant 28, Project Team).

 Others thought that five personnel were required when there was a particularly time-critical case. Consideration of recruitment and rostering aspects (eg, stroke-centric cases and reduced exposure of breadth of clinical case types for paramedics) were also raised – upskilling opportunities and deskilling concerns were identified for those working on the MSU and those at receiving hospitals.

 “*So the general rule of thumb is they love it [working on MSU], absolutely love it, myself included, but still want to maintain their skills elsewhere-- and I feel that the paramedics would say the same. Love it at the time, but if you did it all the time, you’d lose your other clinical skills very, very quickly. And it’s the same for the radiographers”* (Participant 26, Project Team Member AND Clinician on MSU).

 In addition, operational aspects such as changes to the dispatch protocol (eg, increase accuracy for suspected stroke cases) and the potential for telemedicine options (eg, technology and infrastructure, relevance of Victorian model for other Australian states) were raised.

 “*I think the future would be to expand the service. I think it’s a really helpful thing to do, but I think the main way to make it more sustainable is finding a better triaging system [ESTA call-taker protocol] because I think that’s the main barrier to it being an effective service”* (Participant 27, Clinician on MSU).

 “*The telemedicine facility has got to be utilised. It’s there. It should be built into all the future systems as well because the logic of having a stroke neurologist running around on an ambulance, hoping to see a stroke patient is probably a waste of resource”* (Participant 28, Project Team).

 The importance of multi-organisation communication (eg, sharing of interim results, receiving hospitals to be involved in streamlining MSU patient processes relevant to their hospital) was also identified.

 “*I think feeding back or reporting to each peripheral centre as well, how many cases in their normal catchment were diverted, would be useful”* (Participant 11, Clinician at receiving hospital).

 While some considered the next stage to be the scanner in a helicopter, another considered expanding the fleet with stroke clinicians in conventional ambulances.

## Discussion

 To the best of our knowledge, this is the first multi-stakeholder implementation evaluation focusing on the sustainability of the MSU model of care. Overall, participants wanted to continue working within the MSU model and could identify advantages for patients and clinicians. Important factors associated with wanting to continue working within the MSU model were identified from two theories: being able to identify advantages of the MSU to their daily work (performance expectancy; UTAUT), and not experiencing pressures and tensions, along with feeling working competently as part of the MSU team (intrinsic motivations; SDT). Particular insights included that advantages extended beyond patient care to include clinicians, while disadvantages required systemic-level changes. Importantly, the groups working with the MSU had a somewhat different experience compared to those who were working on the MSU or in broader operational roles. Points raised to consider for the MSU’s future addressed each aspect of MSU operations: vehicle design, team composition, dispatch protocol, recruitment, and rostering, multi-organisation integration, potential for telemedicine, working relationships and processes with receiving hospitals.

 Reporting that working on the MSU program was beneficial in daily work was the most strongly associated item with wanting to continue working with the MSU model in the future. The main advantages of the MSU identified were improvements in patient care, improved clinical processes and patient outcomes. These included faster scan, diagnosis and treatment times.^7,11^ These findings are unsurprising given the profile of MSUs delivering pre-hospital care for time-critical condition of stroke, however it is important to note that such advantages were identified across all groups, particularly with co-dispatched paramedics. This is important as some paramedics had expressed initial concerns about delays in transporting patients while waiting for the MSU.^18^ Improved diagnosis and treatment times have been partially explained by the increased efficiencies between pre-hospital paramedics and hospital clinicians.^7^ While the reduction in time to thrombolysis treatment is described as “the essence of MSUs,” Audebert and colleagues identified additional advantages including stroke-expert triage and transport to hospitals with stroke units.^30^ Improved knowledge was also frequently cited in our results, ranging from stroke-specific clinical knowledge for paramedics working on and with the MSU (eg, assessment, non-stroke management) and hospital clinicians’ greater understanding of pre-hospital conditions and stroke care. This information can be used to support the MSU model and recruitment of future MSU team members.

 Feeling as though participants were working competently as part of a team without making individual performance comparisons was also a key predictor of wanting to work on/with the MSU program in future. Given the complexities in the delivery of the MSU model (eg, collaborations across and within multiple organisations and stakeholders) and the interdisciplinary approach required for stroke care in time critical acute conditions, working as a team is an essential factor in delivering optimal care within the MSU model. Stakeholder inclusion and collaboration throughout development and operations has previously been identified as essential for success.^12^ Additional advantages of the MSU model were aspects of team work including the interdisciplinary working relationships and working with other health professionals or partner organisations. Working on and with the MSU was described as a learning environment for all. These results suggest that an integrated team approach will be a particularly important component to the MSU program’s ongoing success.

 Importantly, all groups were able to identify advantages of the MSU and wanted to continue working within the MSU program in future. However, differences between groups were most visible when considering those working with the MSU, and to our knowledge, these have not been previously identified. In our study, these participants were predominantly co-dispatched paramedics (approximately 80% of sub-sample) with the balance clinicians at receiving hospitals. Results indicated that this group had a somewhat different experience than those working on the MSU (eg, paramedics, radiographers, stroke clinicians) or working operationally (eg, project leads, executives). Differences included reporting lower perceived organisational readiness and not having a choice about working with the MSU. Importantly, reports of not being familiar with the MSU procedure or protocol were indicated by co-dispatched paramedics. For those considering implementing MSUs in future, these results indicate the importance of working relationships between those working on developing the MSU and working on the MSU with those from receiving hospitals. The pressure of having the MSU go live was reported as contributing to the implementation^18^ and in addition, the MSU service was initially a pilot project, limiting the focus on exploring in detail broader integration, for example, establishing changes to receiving hospital’s processes. Additionally, communication across the AV workforce is complex due to the high number, distribution and rotation (roster) of personnel. There are almost 5000 full-time equivalent personnel with >4000 on-road clinical staff in 2018-2019,^31^ across almost 300 branches across Victoria.^32^ There are approximately 2900 full-time equivalent on-road clinical staff in 102 branches within metropolitan Melbourne, with 1 in 5 paramedics on leave at any one time. Recall of early communications about changes may have reduced by the time the MSU was operational and the arrival of patients at receiving hospital EDs was occurring. Limited exposure to the MSU was noted by some. Personal experiences and exposure to technology-based clinical changes can lead to changes in initial attitudes and beliefs.^33^ As such, additional targeted facilitation and information dissemination after the MSU was operational could have been beneficial. Achieving blanket communication for all may be more difficult where MSUs operate in areas with multiple emergency service organisations and/or certain rostering profiles.

 Although those working with the MSU indicated that their role had not changed as a result of working with the MSU model of care, they also reported a number of role-relevant disadvantages, including initially experiencing difficulties working with multiple teams and disciplines at a patient scene. These concerns could have been exacerbated by the change to paramedics’ usual brief of ‘getting a patient with suspected stroke to hospital as quickly as possible’ (ie, ‘load and go’) to having to wait with the patient on scene for the MSU arrival. In the early period of the MSU, having to wait for the somewhat unknown MSU contrasted with their previous well-established practices of rapidly identifying and transporting patients with suspected stroke to hospital, which are incorporated in their performance metrics.^31^ This negative perception of ‘waiting’ illustrates the importance of providing sufficient information to explain the benefits of waiting for the MSU on-scene. Improvements in onset-to-treatment times have been reported for cases with up to an average of 18 minutes MSU travel time (from where located when dispatched).^34^ Therefore, regular ambulance crews can be advised that waiting for the MSU to arrive can yield improved treatment times or indeed, meeting the MSU partway on route can be undertaken. The low exposure to MSU operations in the field combined with the change in paramedics’ protocol is an important one to address as co-dispatching the MSU with a standard ambulance is the typical operational model internationally.^30^ This initial confusion regarding the MSU role therefore extended beyond immediate patient care to operational performance issues, such as those associated with extended on-scene metrics for AV. This finding illustrates the importance of communication and adjusting operational metrics and systems to incorporate the changes in the clinical care journey within the broader healthcare system.

 Understanding negative perceptions of the MSU are important to explore and address, as relevant. A disadvantage predominantly identified by co-dispatched paramedics was the availability of the MSU; that is, only one vehicle, with limited hours and operational geographical area. As this disadvantage was identified mostly by those working with the MSU, this suggests that despite some difficulties, expanding the MSU’s availability would be considered beneficial. Limited operating hours (ie, not 24/7) and catchment areas (eg, 20 km radius or between 15 and 20 minute response radii) are usual operational limitations of MSUs.^10^ A disadvantage predominantly identified by clinicians at receiving hospitals was a suboptimal handover or communication process. Streamlined communication systems between MSUs and emergency medical services have been previously identified as important, but difficult, to deliver.^12^ Having a multi-organisational communication platform would support shared access to documented information, bi-directional communication when clarifications are required, along with receiving and incorporating MSU clinical notes into hospital systems. The importance of communication for patient handovers has been previously identified, including from paramedics to ED staff in other research^35,36^ with results from previous non-MSU studies showing miscommunication is commonly associated with hospital adverse events.^37-39^ Health Insurance Portability and Accountability Act compliant communication tools (eg, smartphone apps) may address these communication concerns, which existed prior to, but are exacerbated by, the availability of the MSU.

 Results across a number of themes and groups identified variation in understanding current operations. For example, some paramedics thought that there were different employee advantages when working on the MSU (eg, access to overtime on longer days) with risks to self (eg, not meeting key performance indicator of on-scene times) and patient risks (eg, delayed transport to hospital) from waiting for the MSU. These results suggest the importance of appropriate systemic changes and communication to support the sustainability of this new model. The variations in perceptions and understanding may be explained by the difficulty of communication across organisations, or that changes (or not) to individuals are incorporated beyond the initial MSU implementation.

 Despite telemedicine in MSUs being long considered,^7^ and AV having an acute stroke telemedicine service across regional Victoria^40^ as part of their business-as-usual services, telemedicine within the Melbourne MSU is yet to be established. Variations in patient care locations (eg, patient’s initial assessment in home, not within vehicle) means that telemedicine infrastructure needs to be mobile, not only built into the vehicle.^20^

 Funding the MSU was raised by only a few participants, including if it was a cost-effective option and the lost opportunity of resources for elsewhere, yet is a particularly important aspect for the ongoing operations of MSUs.^12^ Funding difficulties and sources may vary from an initial pilot project to an ongoing business-as-usual model. As the MSU becomes further integrated into current systems, and processes are streamlined, the initial attraction for some (not all) may wane (eg, early adopters).^41^ For example, the MSU becomes more business-as-usual for stroke care and for some, may no longer considered a novel nor exciting opportunity. This integration of the model and reduction in novelty within stroke care, may be particularly relevant for early adopters who look to the next novel opportunity. Those initially keen to staff the MSU model may have less interest in doing so. Ongoing changes (eg, remote neurologist and telemedicine, helicopter model) may mitigate some of this, but in time, the day-to-day business of the MSU may attract and retain different individuals than during the initial implementation phase (eg, late majority, laggards).^41^ This adaptation to novel interventions is found in other areas (eg, telemedicine), requiring careful handover to ensure smooth functioning as adaptations (eg, additional MSUs) are integrated. While some factors identified can be addressed by further communication (eg, sharing of preliminary results, clarification of employee benefits) and collaboration (eg, working more closely with receiving hospitals individually, providing updated training for new team member on-boarding), others need systemic considerations (eg, shorter roster duration for paramedics on the MSU to allow broader clinical case exposure, communication methods across organisations, multi-organisational documentation). These aspects become more important as the number and frequency of patients to receiving hospitals increases and the MSU model is extended. Results from economic evaluations will also contribute to optimal staffing model/s.^15^

###  Strengths, Limitations and Future Research

 There are a number of strengths to this MSU process evaluation. This study was undertaken by those associated with but separate from the operations of the Melbourne MSU, and incorporated stakeholders from across all roles and organisations involved in its development and operations. This external evaluation team provided a more objective approach than previous reports, reducing researcher and participant bias. Participant inclusivity provided unique insights to differences amongst those involved in the implementation of the MSU model. Our mixed-method approach including a theory-based questionnaire, provided the opportunity to identify predictive factors for the sustainable work practices for the MSU along with specific details to augment findings from our in-depth interviews and focus groups. However, there are several limitations to consider. The first and foremost is that these data have been drawn from one MSU service implemented in Australia. Factors raised may be due to the specific vehicle design used (eg, size and layout of truck), the combination of team members deployed or the geographic area. Generalisability to MSUs in other healthcare and social contexts requires further exploration. For example, the Melbourne MSU works with a single emergency care provider which services the entire state. Previous work has been completed predominantly in Berlin, Germany and Houston, USA. Those areas where multiple providers operate and different regulations exist may have different experiences.^42^ While the implementation and operational specifics may differ, our results can prompt consideration of the relevance of such factors and how to incorporate them locally.^12^ Our participant sample had only a small number of personnel in executive- or project lead-only roles without any clinical experience, and therefore precluded separate analyses to be undertaken with this further sub-group. It could be argued that those working in non-clinical roles could have unique insights. However, the dominance of those with both clinical and operational expertise during the early implementation of the MSU highlights the importance of having the input of those with both perspectives. While some participant groups (eg, ED and radiology representation from receiving hospitals) had few interviewees, input was also provided via survey, and due to self-voluntary participation, participants self-selected and as such, views may not represent all those involved with the MSU service. For example, those with particularly positive or negative views may not have participated. Importantly, these results reflect the perceptions of stakeholders at the time of data collection after two years of operation and may not reflect current perspectives, given changes in experience, operations and available information.

## Conclusion

 The MSU model is a significant disruption to the delivery of usual pre-hospital stroke care, but has provided advantages to patients, clinicians and healthcare organisations. In this first process evaluation to understand the sustainability of the MSU model of stroke care, we have identified that experience of pressure or tension may reduce the availability of future staffing. However, identifying advantages of the MSU in clinical practice could support maintaining a roster for ongoing personnel. Being a competent team member for a cohesive team approach is important both clinically and operationally, and the disadvantages and future considerations identified can be used for adaptations of future versions of the MSU which will help advance this model of care. How additional MSUs operating in the region will impact staffing, operations and advantages across participating organisations awaits further research.

## Acknowledgements

 We would like to acknowledge the members of the MSU Steering Committee and all those involved in this multi-organisation collaboration (RMH, AV, the University of Melbourne, the Florey Institute of Neuroscience and Mental Health, the Stroke Foundation, and RMH Neuroscience Foundation) including Wayne Schocker, Peter Norbury (AV) and Caleb Loo (Monash University) for his assistance setting up the survey in Qualtrics.

## Ethical issues

 Approval for this research was obtained from Melbourne Health Human Research Ethics Committee (HREC/17/MH/375), Ambulance Victoria Research Governance (R18-038) and Monash University Human Research Ethics Committee (20158).

## Competing interests

 HZ has received grants from the Australian Commonwealth Government and the University of Melbourne, and personal fees from National Health and Medical Research Council, Melbourne Health, Boehringer Ingelheim, Medtronic, and Shire. GAD discloses being on the advisory boards of Allergan, Amgen, Bayer, Boehringer Ingelheim, and Servier. SMD discloses grants from the National Health and Medical Research Council and personal fees from Boehringer Ingelheim and Medtronic. SC has received honoraria from Boehringer Ingelheim. SM discloses grants from the National Health and Medical Research Council. SMcG’s organisation has received unrestricted grants from Boehringer Ingelheim not associated with the MSU projects. Others authors declare no conflicts.

## Authors’ contributions

**Conceptualization:** Dominique A. Cadilhac.

**Data curation:** Tara Purvis.

**Formal analysis:** Kathleen L. Bagot, Shaun Hancock.

**Funding acquisition:** Stephen M. Davis, Geoff A. Donnan, Dominique A. Cadilhac.

**Investigation:** Tara Purvis, Shaun Hancock.

**Methodology:** Kathleen L. Bagot, Dominique A. Cadilhac.

**Project administration: **Tara Purvis.

**Resources:** Damien Easton, Dominique A. Cadilhac.

**Supervision:** Dominique A. Cadilhac.

**Validation:** Tara Purvis, Henry Zhao, Skye Coote, Damien Easton, Bruce CV Campbell, Stephen M. Davis, Geoff A. Donnan, Shane Foster, Francesca Langenberg, Karen Smith, Mick Stephenson, Steve Bernard, Sharon McGowan, Bernard Yan, Peter Mitchell, Sandy Middleton, Dominique A. Cadilhac.

**Writing–original draft:** Kathleen L. Bagot.

**Writing–review & editing:** Kathleen L. Bagot, Tara Purvis, Shaun Hancock, Henry Zhao, Skye Coote, Damien Easton, Bruce CV Campbell, Stephen M. Davis, Geoff A. Donnan, Shane Foster, Francesca Langenberg, Karen Smith, Michael Stephenson, Stephen Bernard, Sharon McGowan, Bernard Yan, Peter Mitchell, Sandy Middleton, Dominique A. Cadilhac.

## Funding

 The Melbourne Mobile Stroke Unit and associated projects received funding from the Australian Commonwealth Government, Victorian State Government, Royal Melbourne Hospital Neurosciences Foundation, Stroke Foundation, an NHMRC Program Grant (#1113352), the Florey Institute of Neurosciences and Mental Health, the University of Melbourne, Boehringer Ingelheim, and a private donation. Funding for this evaluation was provided by Melbourne Health from an NHMRC Program Grant (#1113352).

## Supplementary files


Supplementary file 1 contains Figures S1-S2.

## References

[1] Meretoja A, Keshtkaran M, Saver JL (2014). Stroke thrombolysis: save a minute, save a day. Stroke.

[2] Meretoja A, Keshtkaran M, Tatlisumak T, Donnan GA, Churilov L (2017). Endovascular therapy for ischemic stroke: save a minute-save a week. Neurology.

[3] Middleton S, Dale S, Cheung NW (2019). Nurse-initiated acute stroke care in emergency departments. Stroke.

[4] Meretoja A, Strbian D, Mustanoja S, Tatlisumak T, Lindsberg PJ, Kaste M (2012). Reducing in-hospital delay to 20 minutes in stroke thrombolysis. Neurology.

[5] Lachkhem Y, Rican S, Minvielle É (2018). Understanding delays in acute stroke care: a systematic review of reviews. Eur J Public Health.

[6] Fassbender K, Walter S, Liu Y (2003). “Mobile stroke unit” for hyperacute stroke treatment. Stroke.

[7] Walter S, Kostpopoulos P, Haass A (2010). Bringing the hospital to the patient: first treatment of stroke patients at the emergency site. PLoS One.

[8] Fassbender K, Mathur S. The rapidly expanding field of prehospital stroke care In. Mobile Stroke Unit News. Vol 1. Prehospital Stroke Treatment Organisation; 2019.

[9] Coote S, Mackey E, Alexandrov AW (2022). The mobile stroke unit nurse: an international exploration of their scope of practice, education, and training. J NeurosciNurs.

[10] Calderon VJ, Kasturiarachi BM, Lin E, Bansal V, Zaidat OO (2018). Review of the mobile stroke unit experience worldwide. Interv Neurol.

[11] Ebinger M, Kunz A, Wendt M (2015). Effects of golden hour thrombolysis: a Prehospital Acute Neurological Treatment and Optimization of Medical Care in Stroke (PHANTOM-S) substudy. JAMA Neurol.

[12] Parker SA, Bowry R, Wu TC (2015). Establishing the first mobile stroke unit in the United States. Stroke.

[13] Ebinger M, Siegerink B, Kunz A (2021). Association between dispatch of mobile stroke units and functional outcomes among patients with acute ischemic stroke in Berlin. JAMA.

[14] Campbell BCV, Mitchell PJ, Churilov L (2020). Effect of intravenous tenecteplase dose on cerebral reperfusion before thrombectomy in patients with large vessel occlusion ischemic stroke: the EXTEND-IA TNK part 2 randomized clinical trial. JAMA.

[15] Kim J, Easton D, Zhao H (2021). Economic evaluation of the Melbourne mobile stroke unit. Int J Stroke.

[16] Gyrd-Hansen D, Olsen KR, Bollweg K, Kronborg C, Ebinger M, Audebert HJ (2015). Cost-effectiveness estimate of prehospital thrombolysis: results of the PHANTOM-S study. Neurology.

[17] Dietrich M, Walter S, Ragoschke-Schumm A (2014). Is prehospital treatment of acute stroke too expensive? An economic evaluation based on the first trial. Cerebrovasc Dis.

[18] Bagot KL, Purvis T, Hancock S, et al. Interdisciplinary interactions, social systems and technical infrastructure required for successful implementation of mobile stroke units: a qualitative process evaluation. J Eval Clin Pract. 2023. 10.1111/jep.13803. 36648226

[19] Australian Bureau of Statistics. Regional Population, 2019-20 Financial Year. 2021. https://www.abs.gov.au/statistics/people/population/regional-population/2019-20. Accessed December 19, 2021.

[20] Zhao H, Coote S, Easton D (2020). Melbourne mobile stroke unit and reperfusion therapy: greater clinical impact of thrombectomy than thrombolysis. Stroke.

[21] Tong A, Sainsbury P, Craig J (2007). Consolidated criteria for reporting qualitative research (COREQ): a 32-item checklist for interviews and focus groups. Int J Qual Health Care.

[22] Strauss A, Corbin J. Basics of Qualitative Research Techniques. Thousand Oaks, CA: SAGE Publications; 1998.

[23] Venkatesh V, Morris MG, Davis GB, Davis FD (2003). User acceptance of information technology: toward a unified view. MIS Q.

[24] Shea CM, Jacobs SR, Esserman DA, Bruce K, Weiner BJ (2014). Organizational Readiness for Implementing Change: a psychometric assessment of a new measure. Implement Sci.

[25] Ryan RM, Deci EL (2000). Self-determination theory and the facilitation of intrinsic motivation, social development, and well-being. Am Psychol.

[26] Hsieh HF, Shannon SE (2005). Three approaches to qualitative content analysis. Qual Health Res.

[27] Braun V, Clarke V (2006). Using thematic analysis in psychology. Qual Res Psychol.

[28] Pope C, Mays N (1995). Reaching the parts other methods cannot reach: an introduction to qualitative methods in health and health services research. BMJ.

[29] Tzelgov J, Henik A (1991). Suppression situations in psychological research: definitions, implications, and applications. Psychol Bull.

[30] Audebert H, Fassbender K, Hussain MS (2017). The PRE-hospital stroke treatment organization. Int J Stroke.

[31] Ambulance Victoria. Ambulance Victoria 2018-2019 Annual Report. Doncaster: Ambulance Victoria; 2019.

[32] Ambulance Performance and Policy Consultative Committee. Victoria’s Ambulance Action Plan: Improving Services, Saving Lives: Final Report. Melbourne: Victorian Government; 2015.

[33] Marler JH, Fisher SL, Ke W (2009). Employee self-service technology acceptance: a comparison of pre-implementation and post-implementation relationships. Pers Psychol.

[34] Koch PM, Kunz A, Ebinger M (2016). Influence of distance to scene on time to thrombolysis in a specialized stroke ambulance. Stroke.

[35] Solet DJ, Norvell JM, Rutan GH, Frankel RM (2005). Lost in translation: challenges and opportunities in physician-to-physician communication during patient handoffs. Acad Med.

[36] Dawson S, King L, Grantham H (2013). Review article: Improving the hospital clinical handover between paramedics and emergency department staff in the deteriorating patient. Emerg Med Australas.

[37] Leape LL, Berwick DM (2005). Five years after to Err is human: what have we learned?. JAMA.

[38] Sutcliffe KM, Lewton E, Rosenthal MM (2004). Communication failures: an insidious contributor to medical mishaps. Acad Med.

[39] Gawande AA, Zinner MJ, Studdert DM, Brennan TA (2003). Analysis of errors reported by surgeons at three teaching hospitals. Surgery.

[40] Bladin CF, Kim J, Bagot KL (2020). Improving acute stroke care in regional hospitals: clinical evaluation of the Victorian Stroke Telemedicine program. Med J Aust.

[41] Rogers EM. Diffusion of Innovations. 5th ed. New York, NY: Simon & Schuster; 2003.

[42] Rajan SS, Baraniuk S, Parker S, Wu TC, Bowry R, Grotta JC (2015). Implementing a mobile stroke unit program in the United States: why, how, and how much?. JAMA Neurol.

