# Supplementary material for: Sustaining a New Model of Acute Stroke Care: A Mixed-Method Process Evaluation of the Melbourne Mobile Stroke Unit
Source: Int J Health Policy Manag. 2023 Apr 5;12:7716. doi: 10.34172/ijhpm.2023.7716 (PMC10461847; doi:10.34172/ijhpm.2023.7716)
Supplement: Supplementary file 1 — contains Figures S1-S2. [file ijhpm-12-7716-s001.pdf]

Article title: Sustaining a New Model of Acute Stroke Care: A Mixed-Method Process Evaluation of the Melbourne Mobile Stroke Unit

Journal name: International Journal of Health Policy and Management (IJHPM)

Authors' information: Kathleen L. Bagot[1,2], Tara Purvis[2], Shaun Hancock[1], Henry Zhao[3,4,5,6], Skye Coote[3,4], Damien Easton[3,4], Bruce CV Campbell[3,4,6,7], Stephen M. Davis[3,4], Geoff A. Donnan[1,3,4], Shane Foster[6], Francesca Langenberg[3,6], Karen Smith[6,8,9], Michael Stephenson[6,8], Stephen Bernard[6,8], Sharon McGowan[7], Bernard Yan[3], Peter Mitchell[4], Sandy Middleton[10,11], Dominique A. Cadilhac[1,2]*

[1]Public Health and Health Services Research, Stroke, The Florey Institute Neuroscience and Mental Health, Heidelberg, University of Melbourne, Melbourne, VIC, Australia.

[2]Stroke and Ageing Research, Department of Medicine, School of Clinical Sciences at Monash Health, Monash University, Clayton, VIC, Australia.

[3]Department of Neurology, Melbourne Brain Centre, Royal Melbourne Hospital, Melbourne, VIC, Australia.

[4]Department of Medicine, Faculty of Medicine, Dentistry and Health Sciences, University of Melbourne, Melbourne, VIC, Australia.

[5]Austin Health, Faculty of Medicine, Dentistry and Health Sciences, University of Melbourne, Melbourne, VIC, Australia.

[6]Ambulance Victoria, Melbourne, VIC, Australia.

[7]Stroke Foundation, Melbourne, VIC, Australia.

[8]Department of Epidemiology and Preventive Medicine, Department of Paramedicine Monash University, Melbourne, Melbourne, VIC, Australia.

[9]Discipline of Emergency Medicine, University of Western Australia, Perth, WA, Australia.

[10]St Vincent's Health Network Sydney, St Vincent's Hospital Melbourne, Melbourne, VIC, Australia.

[11]Nursing Research Institute, Australian Catholic University, Melbourne, VIC, Australia.

(*Corresponding author: dominique.cadilhac@monash.edu)

Supplementary file 1 contains Figures S1-S2

Figure S1 - Survey content, tailored for different groups

Figure S2 - Individual / Group Semi-structured Interview Schedule

Supplemental Figure 1

Survey content, tailored for different groups (stakeholders)

General questions (asked to all)

• Your organisation:

- Hospital ____________________ (insert name)
- Ambulance Victoria
- Other (specify)

If hospital, your department/unit/team

- Emergency department, Radiology, Neurology, Other (specify)

• Time in current role: years/months

• Which of the following statements best describes your role (tick all that apply)

- Clinician working on the MSU
- Paramedic co-dispatched with the MSU or ESTA dispatcher
- Clinician at hospital receiving MSU patients
- Program Operational Team (Administrator, Project Officer)
- Executive, Organisation Manager

• Select your profession (one option)

MICA paramedic, ALS paramedic, Neurologist, Physician, Registrar, Intern (Resident), Nurse, Manager (e.g. NUM), Radiographer, Other (specify)

• Age

• Gender

Male, Female, Other (Specify)

• Employment status

Full time ongoing, full time fixed term, part time ongoing, part time fixed term, casual, other (specify)

• Are you happy to be contacted to further obtain your views on the MSU through a focus group or 1:1 interview at a time convenient to you? Yes/No

If yes, please provide your contact details to allow us to get in touch with you.

Please note your contact details will be stored separately from your survey responses

If you decide to take part and later change your mind, you are free to withdraw at a later stage

Name/contact number/email

✓ Question asked for particular stakeholder

	Questions	Clinicians working on MSU	Paramedics co-dispatched with the MSU (+ ESTA)	Clinicians at hospitals receiving MSU patients	Program operational team (admin, project officers)	Executives, Organisation managers
**INVOLVEMENT WITH THE MSU program**						
1	Were you involved in the decision making to implement the MSU program? Yes/No	✓	✓	✓	✓	✓
1a	If so, what role did you perform (tick as many as apply):- approved funding, provided authorisation to participate clinically, assigned the project to others to deliver, delivered training/education, talked with others about how the MSU would work, other (specify)	✓	✓	✓	✓	✓
2	Were you involved in the roll-out (implementation) of the MSU program? Yes/No	✓	✓	✓	✓	✓
2a	If so, how were you involved (tick as many as apply)- conducted training/education sessions, attended training sessions, attended information/education, participated in mock patient trials, worked on the MSU during roll out, other (specify)	✓	✓	✓	✓	✓
3	Have you worked clinically on/with the MSU in your role? Yes/No	✓	✓	✓	✓	✓
3a	If yes, approximately how many of your patients have the MSU been engaged with? <10, 10-29, 30-49, 50-99, 100+	✓	✓	✓	✓	✓
4	Were there times when you did not use/work with the MSU even though there was an eligible patient? Yes/No	✓	✓	**x**	**x**	**X**
4a	If yes, describe why- MSU unavailable, technology reasons, patient specific case, other- specify	✓	✓	**x**	**x**	**x**
**AGREEMENT STATEMENT ABOUT THE MSU- (Completely disagree, Strongly disagree, Disagree, Neutral, Agree, Strongly agree, Completely agree, Not relevant to me)**						
5	Working on/with the MSU program has become natural to me	✓	✓	✓	x	x

6	I would like to work on/with the MSU program in the future	✓	✓	✓	x	✓
7	I always tried to engage with the MSU program in my clinical work as required	x	✓	✓	x	x
8	I found working on/with the MSU program interesting (across entire program development)	✓	✓	✓	✓	✓
9	I did not really have a choice about working on/with the MSU program	✓	✓	✓	x	✓
10	I think I did well at working on/with the MSU program, compared to colleagues	✓	✓	✓	✓	x
11	After working on/with the MSU program for a while, I felt competent in my role in relation to the program	✓	✓	✓	✓	x
12	I felt like I was able to work to my full scope of practice while I was working on/with the MSU program	✓	✓	✓	x	x
13	I did not feel at all nervous about working on/with the MSU program as a new model of care	✓	✓	✓	✓	✓
19	I felt pressured while working on/with the MSU program	✓	✓	✓	✓	✓
20	I felt I had the resources necessary to work on/with the MSU program	✓	✓	✓	✓	✓
21	I had the knowledge to work on/with the MSU program	✓	✓	✓	✓	✓
22	My role in working on/with the MSU program was clear	✓	✓	✓	✓	✓
23	Working on/with the MSU program was beneficial in my daily work	x	✓	✓	x	x
24	Learning how to work on/with the MSU program was easy	✓	✓	✓	✓	x
25	I felt my team/organisation were ready to work on/with the MSU program	✓	✓	✓	✓	✓
26	Peers and managers were supportive of my role working on/with the MSU program	✓	✓	✓	✓	x
27	My role has changed as a result of working on/with the MSU program	✓	✓	✓	✓	✓
28	All challenges arising from working on/with the MSU program were managed (regardless of who was responsible for the management)	✓	✓	✓	✓	✓
**Addition Hosp & AV Managers/Area Leaders only**						
29	Staff/colleagues were invested in working with the MSU program	x	x	x	X	✓
30	Staff/colleagues were supported in working with the MSU program	x	x	x	x	✓
**EXPERIENCE WITH THE MSU**						
31	Describe three barriers to working with the MSU program in your role/team/organisation.	✓	✓	✓	✓	✓

32	Describe three benefits of working with the MSU program in your role/team/organisation.	✓	✓	✓	✓	✓
33	Does working with the MSU program differ from working with a regular ambulance (ALS/MICA) Yes/No/Not Applicable	✓	✓	✓	✓	x
33a	If yes, describe	✓	✓	✓	✓	x
34	Do you have any concerns regarding patient safety working with the MSU? Yes/No	✓	✓	✓	✓	✓
34a	If yes, describe	✓	✓	✓	✓	✓
35	Do you believe the MSU program improves patient care compared to usual practice? Yes, No	✓	✓	✓	✓	✓
35a	Please describe	✓	✓	✓	✓	✓
36	Is there anything unclear or uncertain about working on/with the MSU program? Yes/No	✓	✓	✓	✓	✓
36a	Please describe	✓	✓	✓	✓	✓
37	Describe 3 features that were helpful in the implementation of the MSU program	✓	✓	✓	✓	✓
38	3 features to be improved about the implementation	✓	✓	✓	✓	✓
**If you have worked on the MSU - describe your experience working**						
39a	Inside the MSU compared to your usual workplace	✓	x	x	x	x
39b	Out in the community compared to your usual workplace	✓	x	x	x	x
39c	Access to technology compared to your usual workplace	✓	x	x	x	x
39d	With colleagues (e.g. paramedics, other hospital staff) compared to your usual workplace	✓	x	x	x	x
40	Do you have any other thoughts or ideas you would like to share about the implementation and proposed operations of the MSU that you think are important for us to know?	✓	✓	✓	✓	✓

Supplemental Figure 2

Individual / Group Semi-structured Interview Schedule

(includes leading question, and potential prompts)

Preamble (to include points following)

- From Monash University, commissioned to independently undertake an evaluation of the Mobile Stroke Unit implementation.

- Important part is to get an understanding of the impact of the MSU from multiple perspectives.

- Interested in your feedback and experiences related to the operational and organisational aspects of the MSU, to ultimately improve the service planning and inform the development of future similar services.

- Record the interview- if ok. Can stop at any time

- All responses will be anonymous- no names recorded

- Can view transcript at end if you like

- Questions before we start?

Can you describe your experience working with the MSU

- *Was it easy, helpful, hindrance*
- *Anything confusing?*

Did you value/understand the reason for the MSU – sense of purpose?

- *Think it was a good idea*

How did the introduction of the MSU affect your work practices/procedures?

- *the way you work (Patterns of workflow- clinical decision making)*
- *the way your area functions*
- *Technical- technology, work settings*
- *Your working environment- Physical*
- *Were there deviations from protocol*

Has the introduction of the MSU affected (relationships):

- *Working relationships- social interactions (communications)—people, tasks, relationships- who and how you work with others*
- *(Neurologist/stroke nurse on board)*

Was there support to incorporate the changes related to the MSU within your role?

- *Did you receive any training related to this?-*
- *Did you have the knowledge to perform your role*
- *Resources available?*
- *Were you confident of your ability to perform the required role*

How different was it from your experience working prior to the MSU

- *Differences, similarities? Compatible with usual work practices*
- *Time taken to assess/diagnose/treat/transfer, decision making process- easier/harder*

Have you had any feedback related to your role/the MSU? Are you aware of the effect the MSU has had?

Any specific IT issues?

Future

Were all aspects of the implementation followed by all in your team?

Do you have any suggestions for improvements/requirements for future long-term use

- *Rollout process*
- *Training requirements*
- *Information/education*

Recommended for use in other settings? Why/why not?

Wrap up
